# Red Blood Cell Anchoring Enables Targeted Transduction and Re‐Administration of AAV‐Mediated Gene Therapy

**DOI:** 10.1002/advs.202201293

**Published:** 2022-07-03

**Authors:** Zongmin Zhao, Jayoung Kim, Vinny Chandran Suja, Neha Kapate, Yongsheng Gao, Junling Guo, Vladimir R. Muzykantov, Samir Mitragotri

**Affiliations:** ^1^ John A. Paulson School of Engineering and Applied Sciences Harvard University Cambridge MA 02138 USA; ^2^ Wyss Institute for Biologically Inspired Engineering at Harvard University Boston MA 02115 USA; ^3^ Harvard‐MIT Division of Health Sciences and Technology Massachusetts Institute of Technology Cambridge MA 02139 USA; ^4^ Department of Systems Pharmacology and Translational Therapeutics and Center for Translational Targeted Therapeutics and Nanomedicine Perelman School of Medicine University of Pennsylvania Philadelphia PA 19104 USA; ^5^ Present address: Department of Pharmaceutical Sciences College of Pharmacy University of Illinois Chicago Chicago IL 60612 USA

**Keywords:** adeno‐associated virus, gene therapy, immunomodulation, neutralizing antibody, red blood cell hitchhiking

## Abstract

Adeno‐associated virus (AAV)‐mediated gene therapy is a promising therapeutic modality for curing many diseases including monogenic diseases. However, limited tissue‐targeting and restricted re‐administration due to the vector immunogenicity largely restrict its therapeutic potential. Here, using a red blood cell (RBC) as the carrier vehicle for AAV is demonstrated to improve its tissue‐targeted transduction and enable its re‐administration. Anchoring AAV to the RBC surface minimally affected its infectability toward endothelial cells. Meanwhile, AAV anchored onto RBCs is predominantly delivered to and shows efficient transduction in the lungs by virtue of the biophysical features of RBCs. RBC‐anchored AAVs lead to a four‐ to five‐fold enhancement in target gene expression in the lungsas compared to free AAVs following a single‐ or dual‐dosing regimen. While RBC anchoring does not prevent the induction of adaptive immune responses against AAV, it results in successful transgene expression upon re‐administration following prior AAV exposure. The ability to re‐administer is partially attributed to the delayed and reduced AAV neutralization by neutralizing antibodies, resulting from the combination of limited exposure of physically confined AAVs and the short time required to reach the lungs. This study's findings suggest that the RBC‐mediated approach is a promising strategy for repetitive, targeted AAV gene therapy.

## Graphical abstract

Clinical implementation of adeno‐associated virus (AAV)‐mediated gene therapy is hindered by its limited tissue‐specificity and inability to re‐dose. Physically anchoring AAV onto RBC surfaces is demonstrated toenable dual functions of contact‐enabled targeting of the lung tissue and kinetics‐based immune evasion by delayed neutralizing antibody detection. This approach represents an effective strategy to overcome anti‐AAV immunity for repetitive, targeted AAV gene therapy.

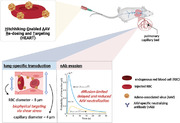

## 1 Introduction

Gene therapy is a potent strategy to treat diseases of genetic origin by correction of an endogenous or expression of an exogenous gene. Among viral vectors that have been investigated as potent gene carriers, adeno‐associated virus (AAV) in particular is most commonly used for in vivo applications in part because distinctive binding motifs on the host cell's AAV receptor allows different levels of serotype‐specific organ tropism and transduction efficacy.^[^ ^1^, ^2^, ^3^, ^4^, ^5^ ^]^ Moreover, AAV vector genomes have been observed to remain in their non‐integrating circular episomes in many tissue types, which prevents adverse event rising from insertional mutagenesis.^[^ ^6^, ^7^ ^]^ However, this, along with AAV capsid‐specific immune responses,^[^ ^8^, ^9^ ^]^ leads to non‐permanent transduction of the delivered gene, especially in dividing cells, thus requiring injection of high dose or repeated administrations to achieve long‐term stable expression of the therapeutic protein.

The immune response from the host upon administration of non‐modified recombinant AAV presents a significant barrier to the multiple dosing regimen.^[^ ^10^, ^11^ ^]^ Specifically, the generation and binding of neutralizing antibody (nAb) effectively prevent AAV entry into target cells. Previous studies have demonstrated a nearly complete blocking of transgene expression from repeated administration.^[^ ^12^, ^13^, ^14^ ^]^ Current approaches to address the challenge in the production of and immune recognition by nAb most commonly employ strategies to re‐engineer the AAV capsid.^[^ ^15^, ^16^, ^17^ ^]^ This includes the use of empty capsids as decoys,^[^ ^18^, ^19^ ^]^ mutation of conserved residues targeted by nAb,^[^ ^20^ ^]^ combination of different serotypes to yield chimeric vectors,^[^ ^21^ ^]^ directed evolution from a large structurally‐guided library of AAV capsids through a selective pressure^[^ ^22^, ^23^ ^]^ and chemical modifications, such as surface coating with polyethylene glycol or Lipofectamine‐DNA oligonucleotides.^[^ ^24^, ^25^ ^]^ However, complete immune evasion has proven to be extremely difficult to achieve; especially, overcoming an existing immune response (the presence of nAbs) to enable AAV re‐dosing remains a significant challenge. Therefore, there is an urgent need for novel engineering of AAV delivery strategies to evade nAb.

**Figure 1:**
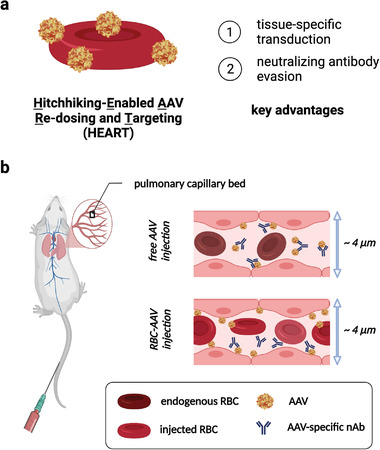
Schematic showing the principle of AAV immune evasion and targeted transduction mediated by HEART (RBC anchoring). Schematic showing a) the design of HEART and b) the process of lung targeting and immune evasion of RBC‐AAVs enabled by HEART as they travel through the narrow lung capillaries.

To solve this challenge, in this work, we demonstrate a new red blood cell (RBC)‐mediated strategy that involves the anchoring of AAVs to the surface of RBCs, which we refer to as Hitchhiking‐Enabled AAV Re‐dosing and Targeting (HEART), to effectively evade nAbs against AAVs and allow targeted tissue transduction (**Figure** **1**a). We have recently developed a novel lung‐targeting drug delivery strategy using an RBC hitchhiking technology.^[^ ^26^, ^27^, ^28^ ^]^ We demonstrated that nanotherapeutics loaded on the surface of RBCs are specifically dislodged in the lung capillary bed during the pulmonary first‐pass following intravenous injection due to mechanical shear.^[^ ^26^, ^27^, ^28^ ^]^ Building upon the lung‐targeting advantage of this technology, we hypothesize that physical confinement of therapeutics to RBC membrane can significantly delay the recognition by the immune system. Of particular interest to this new kinetics‐based paradigm of immune evasion is RBC‐anchored AAVs (RBC‐AAV), as they require less than a minute of protection against the nAbs in the bloodstream until getting deposited in the target organ of interest. We thus engineered HEART as a novel AAV delivery system utilizing RBC as the carrier based on the hypothesis that physically constraining AAV on a large surface area of RBC membrane exhibits dual functions of contact‐enabled targeting of the lung tissue as well as kinetics‐based immune evasion by delayed nAb detection (Figure 1b and Figure S1, Supporting Information). We show that HEART enables re‐dosing of AAVs and lung‐specific transduction in the presence of anti‐AAV nAbs.

## 2 Results

### 2.1 AAV Efficiently Anchors onto RBCs while Minimally Impacting its Infectability

**Figure 2:**
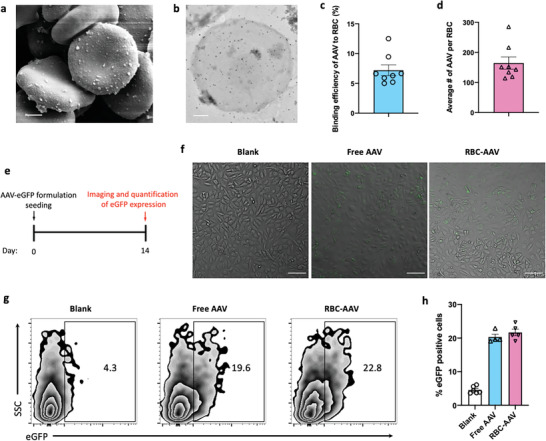
Characterization of AAV anchoring onto RBCs via a polyphenol‐mediated method. a–d) Qualitative and quantitative characterization of the binding of AAV on RBCs. a) Representative SEM images of RBCs carrying AAV. Scale bar: 1 µm. b) Representative TEM images of AAV anchored onto RBCs. Scale bar: 1 µm. c) Binding efficiency of AAV onto RBCs. d) Loading capacity (# of AAV per RBC) of AAV on RBCs. e–h) AAV anchoring onto RBCs minimally affected its infectability. e) Schematic showing the experimental design for measuring the AAV infectability toward EA.hy926 endothelial cells. f) Confocal laser scanning microscopic (CLSM) images of EA.hy926 cells on day 14 at an AAV to EA.hy926 cell incubation ratio of 10 000: 1. Green fluorescence indicates eGFP expression. Scale bars represent 100 µm. g) Representative flow cytometry plots showing the expression of eGFP in EA.hy926 cells on day 14 at an AAV to EA.hy926 cell incubation ratio of 10 000:1. h) Percentage of eGFP positive EA.hy926 cells analyzed by flow cytometry corresponding to (g). Data in (c,d,h) are presented as mean ± sem.

We first established a method to efficiently anchor AAV to RBCs. Adeno‐associated viruses of serotype 9 (AAV9) is used in this work because it represents a clinically relevant AAV serotype that is extensively used in approved products and active clinical trials.^[^ ^5^ ^]^ AAV9 is simply referred to as AAV hereafter. To test spontaneous binding of AAV on the surface of RBCs, we incubated AAV with RBCs at room temperature for 40 min based on a previously described protocol.^[^ ^27^ ^]^ AAVs have a naturally low binding efficiency to RBCs of 1.6% (Figure S2, Supporting Information). We then explored a polyphenol‐mediated approach to improve AAV binding to RBCs.^[^ ^29^ ^]^ We have demonstrated in our previous studies that the polyphenol, tannic acid in this case, modifies biomolecules including AAVs, and facilitates their binding to cell surfaces in the presence of iron.^[^ ^29^ ^]^ Scanning electron microscopy (SEM), transmission electron microscopy (TEM), and real‐time quantitative polymerase chain reaction (RT‐qPCR) analyses confirmed that AAVs were anchored onto RBCs using this approach (**Figure** **2**a,b) at a relatively high efficiency of 7.2% (Figure 2c). Further quantification revealed that on average ~165 AAV particles were anchored onto one RBC at an AAV/RBC incubation ratio of 2300:1 (Figure 2d). AAV anchoring at this loading capacity did not damage RBCs, as indicated by the absence of hemolysis, agglutination, and microscopic morphological changes of RBCs (Figure S3, Supporting Information). Infectability of RBC‐anchored AAV was confirmed using AAVs carrying a green fluorescent protein (eGFP) gene (AAV‐eGFP) in a 2D endothelial cell culture model (Figure 2e). AAV that was anchored onto and subsequently detached from RBCs maintained its capability to transduce and express eGFP in EA.hy926 endothelial cells (Figure 2f and Figure S4, Supporting Information). Flow cytometry further confirmed that the detached AAV from RBCs exhibited similar eGFP expression in EA.hy926 endothelial cells as the free AAV at an equivalent dose (Figure 2g,h and Figure S5, Supporting Information), suggesting AAV anchoring onto RBCs via the polyphenol‐mediated approach does not adversely impact its transduction capability.

### 2.2 RBC Anchoring Delivers AAV Specifically to the Lung and Leads to Targeted Transduction

**Figure 3:**
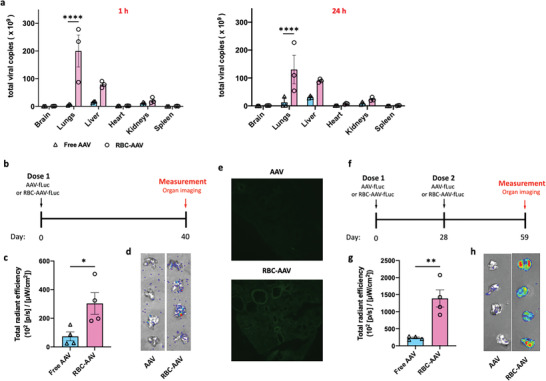
RBC anchoring‐ enabled delivery of AAVs and improved gene expression in the lungs. a) Biodistribution of AAV formulations at different time points after intravenous administration. b–d) Luciferase expression in organs following a single‐dose intravenous administration of AAV‐fLuc formulations. b) Schematic showing the schedule of the study. c) Quantification of the luciferase gene expression on day 40 as indicated by bioluminescence. d) IVIS images of mouse lungs on day 40 following the administration of AAV‐fLuc formulations. e) Fluorescence microscopic images of lung tissues on day 40 following a single‐dose intravenous administration of AAV‐eGFP formulations. Green fluorescence indicates eGFP expression. Scale bars: 250 µm. f–h) Luciferase expression in mouse organs following two doses of intravenously administered AAV‐fLuc formulations. f) Schematic showing the schedule of the study. g) Luciferase gene expression in the lungs on day 59 as quantified by bioluminescence. h) IVIS images of mouse lungs on day 59 following the administration of AAV‐fLuc formulations. Data in (a,c,g) are presented as mean ± sem. Significantly different in (a,c,g) as determined by student's *t* test: * *p* < 0.05, ** *p* < 0.01, and **** *p* < 0.0001.

Our previous studies demonstrated that polymeric nanoparticles adsorbed on the RBC surface are delivered to the lung via a physical mechanism.^[^ ^27^, ^28^, ^30^ ^]^ This lung‐targeted delivery originates primarily from the biophysics of RBCs, where nanoparticles are mechanically dislodged and transferred to lung endothelium as RBCs squeeze through the narrow lung capillaries.^[^ ^27^, ^28^, ^30^ ^]^ Consistent with our previous findings, biodistribution studies confirmed that unlike free AAV which primarily localized in the liver after intravenous administration (**Figure** **3**a), RBC‐anchored AAV deposited primarily in the lungs with 53% and 34% of the injected AAV accumulating in the lung tissue at 1 and 24 h after administration, respectively. AAV accumulation closely matched with its transduction and gene expression assessed using AAV carrying a firefly luciferase (fLuc) gene (AAV‐fLuc) (Figure 3b). RBC‐AAV‐fLuc led to a significantly higher (approximately fourfold enhancement) luciferase gene expression in the lungs as compared to the free AAV‐fLuc 40 days after a single‐dose administration (Figure 3c,d). In addition, anchoring of AAV to RBCs did not significantly alter the luciferase expression in other tested organs apart from the lungs (Figure S6, Supporting Information). In a separate study in which AAV carrying a gene construct encoding eGFP (AAV‐eGFP) was dosed according to the same schedule as in Figure 3b, the lung histological imaging data (Figure 3e) further confirmed the enhanced gene expression with RBC‐AAV in the single‐dose regime. To assess the ability to prolong gene expression, two doses of AAV‐fLuc formulations were intravenously administered on days 0 and 28, and the luciferase gene expression was measured on day 59 (Figure 3f). In the dual‐dose regime, RBC‐AAV further enhanced the gene expressions in the lungs and other major organs 31 days after the second dose as compared to the free AAV (Figure 3g,h and Figure S7, Supporting Information). Particularly, quantification data (Figure 3g) revealed that the RBC‐AAV approach led to an approximately fivefold enhancement in the luciferase gene expression in the lungs. Overall, the biodistribution and gene expression data, combined, revealed that RBC‐anchoring enabled the delivery of AAV specifically to the lungs which in turn resulted in targeted gene expression in the lung tissue. The biocompatibility of HEART was assessed by blood chemistry, hematological analysis, and blinded histological evaluation of major organs post formulation administration. Blood chemistry and hematological analysis data shown in Figures S8 and S9, Supporting Information, suggested that HEART did not lead to significant changes in most of the tested markers as compared to the untreated group. Following the administration of HEART, the creatinine level and platelet number were significantly changed on Day 1 but returned to the similar level on Day 7 as compared to the untreated group. Further, no detectable toxicity from HEART was noted in the tissue based on several criteria including inflammation, fibrosis, ulceration, necrosis, edema, and degeneration (Figure S10, Supporting Information).

### 2.3 RBC Anchoring Enables AAV Re‐Dosing

**Figure 4:**
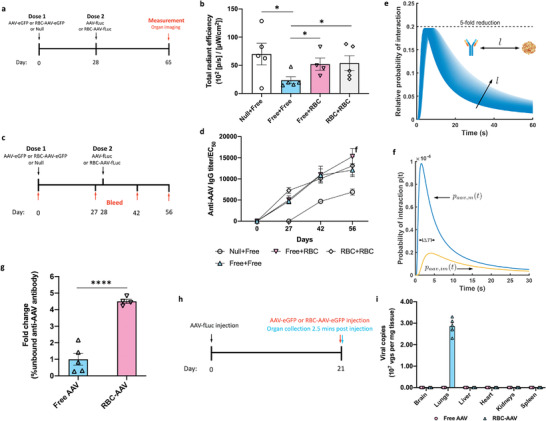
RBC anchoring‐enabled re‐dosing of AAV‐mediated gene therapy and its mechanism. a) Schematic showing the schedule of the study to test the re‐dosing potential of AAV. b) Luciferase gene expression level in the lungs 37 days after the 2nd dose of AAV formulations as measured by bioluminescence. c,d) Study of the humoral immune response (anti‐AAV nAb generation) induced by free AAVs or RBC‐AAVs. c) Schedule of the study. d) Anti‐AAV IgG nAb titer (EC_50_) as quantified by ELISA. e,f) Simulation showing the interactions between nAbs and free AAVs or RBC‐AAVs. e) Relative probability of interaction between an immobilized AAV and neutralizing antibodies as compared to a free AAV and neutralizing antibodies. The parameter *l* is the initial separation between an AAV and a nAb. To show the effect of the initial separation we varied the initial separation by 50% from a baseline case of *l*_aav;im_ = 1000 (*R*_aav_ + *R*_nab_). f) The probability density functions for the baseline case when AAV is mobile *p*_aav;m_(*t*) and when immobile *p*_aav;im_(*t*). Δ*T* denotes the time shift in the peak probability of interaction across the two cases. g) Comparison of the binding of anti‐AAV antibodies to free AAV or RBC‐AAV in vitro. The fold‐change of percentage of unbound antibody for the RBC‐AAV group as compared to the free AAV group was shown. h,i) Study of the biodistribution of free AAVs and RBC‐AAVs 2.5 min after intravenous administration in vaccinated mice. g) Schedule of the study. h) The number of AAVs in different organs as quantified by RT‐qPCR. Data in (b,d,g,i) are presented as mean ± sem. Statistical significance in (b,g) was determined by one‐way ANOVA followed by Tukey's HSD test and student's *t* test, respectively: * *p* < 0.05, **** *p* < 0.0001.

We next investigated the ability of RBC‐AAV to modulate the immune response for successful AAV re‐administration (**Figure** **4**a,b). For this purpose, two different AAVs that carried two different gene constructs encoding either eGFP or fLuc were used. Specifically, AAV‐eGFP was used in the first dose while AAV‐fLuc was used in the second dose. The first dose of AAV‐eGFP induces an immune response against the AAV viral capsid but insensitive to the gene it carries, which allows the expression level of luciferase from the second dose with AAV‐fLuc to be an indicator of the re‐dosing potential of AAVs (Figure 4a). Null group comprised animals without any AAV‐eGFP exposure, and thus no prior immune response against AAV was generated. Luciferase expression level in the lungs in the Free + Free group significantly dropped (a threefold reduction) as compared to that in the Null + Free group, suggesting the immune response dramatically inhibited the re‐dosing potential of free AAV (Figure 4b). In stark contrast, RBC anchoring (Free + RBC and RBC + RBC groups) elevated the luciferase gene expression to a level that is comparable to that of the Null + Free group, indicating that RBC anchoring could navigate the immune response and enable the re‐administration of AAV (Figure 4b). A single‐dose of free AAV (Null + Free group) led to the transduction and luciferase expression mainly in the liver (Figure S11, Supporting Information), which is consistent with the biodistribution data (Figure 3a), where free AAV was mostly detected in the liver following intravenous administration. In the re‐dosing regimen of free AAV (Free + Free group), the luciferase gene expression in all organs was dramatically inhibited, most likely owing to the presence of an induced immune response against AAV (Figure S11, Supporting Information). Interestingly, when using RBC‐AAV as the second dose (Free + RBC and RBC + RBC groups), the highest luciferase expression was observed in the lungs rather than in the livers, although the overall luciferase expressions in all organs are lower as compared to the Null + Free group (Figure S11, Supporting Information). These data evidently revealed that in the presence of an existing immunity against AAV, RBC anchoring could enable targeted transduction of the lung tissue with the AAV vector.

### 2.4 RBC‐AAV Re‐Dosing Is Enabled by a Kinetics‐Based, Diffusion‐Limited Neutralizing Antibody Evasion Mechanism

We next sought to uncover the underlying mechanism of successful AAV re‐administration enabled by the RBC anchoring. First, we performed a series of studies to determine the direct influence of RBC anchoring of AAV on the induction of the humoral and cellular immune responses as measured by anti‐AAV nAb levels and immune cell populations after AAV administration (Figure 4c,d). No statistical difference was found in the nAb levels of animals treated with RBC + RBC compared to Free + Free or Free + RBC animals. In addition, following the second dose on days 42 and 56, the administration of free AAV (Free + Free group) and RBC‐AAV (Free + RBC and RBC + RBC groups) led to similar AAV nAb titers (Figure 4d). Overall, the time‐course data of the anti‐AAV antibody titers revealed that anchoring AAV to RBCs did not significantly impact the humoral immune response. While the induction of anti‐AAV antibodies is most likely the reason for reduced luciferase expression observed in the RBC‐AAV (Free + RBC and RBC +RBC) groups than in the Null + Free group shown in Figure S11, Supporting Information, its effect in the expression level in the lungs was still minimal. Immune cell profiling studies were performed to assess whether cellular immune responses are affected by the RBC anchoring (Figure S12a, Supporting Information). T cell response in the lungs 31 days after AAV administration, as assessed by CD4 and CD8 cell counts, was comparable in the AAV and RBC‐AAV groups (Figure S12b, Supporting Information). No significant differences were found in the immune cells in either spleen or blood as measured using CD4 T cells, CD8 T cells, IFN+ CD8 T cells, central memory T cells, effector memory T cells, and regulatory T cells (Tregs) (Figures S13 and S14, Supporting Information). The immune cell profiling data in the lungs, spleens, and blood consistently revealed that anchoring AAV to RBCs couldn't alter the cellular immune response.

We then hypothesized that RBC‐AAV re‐dosing is mediated by a kinetics‐based nAb evasion, where immobilization of AAVs on RBC membrane sufficiently delays the recognition by circulating anti‐AAV nAbs until the AAVs are sheared‐off and deposited in the lungs. To test this hypothesis, we first performed a simulation of the association between nAbs and AAVs (either free or anchored onto RBCs).^[^ ^31^ ^]^ Simulations revealed that the anchoring of AAVs to RBCs reduced the likelihood of nAbs encountering AAVs (Figure 4e,f). Immobilizing AAVs on RBCs inhibits their diffusion and thus reduces the net diffusivity and increases their statistical distance from nAbs. Simulations predicted a fivefold reduction in the peak probability and a 2.5‐fold increase in the time corresponding to the peak probability of interaction (Figure 4e,f). Moreover, our in vitro binding assay data demonstrated that as compared to the free AAVs, anchoring AAVs to RBCs led to a fourfold reduction in the binding between AAVs and anti‐AAV antibodies 15 min post incubation (Figure 4g). Importantly, the delay in the association between nAbs and AAVs anchored onto RBCs may create a sufficient time window for the delivery and deposition of AAVs to the lungs. To test this possibility, we conducted a biodistribution study at a very early time point (2.5 min) following the AAV formulation administration in mice vaccinated with AAV to generate pre‐existing AAV nAbs (Figure 4h,i). No detectable AAVs were seen in any of the tested organs for the free AAV injections. However, a substantial number of AAVs was seen in the lung tissues for the RBC‐AAV group even at a short time of 2.5 min. No AAV was seen in other organs (Figure 4i). These data supported the hypothesis that the delay in the recognition of AAV by nAbs enabled the delivery and deposition of AAV particles in the target lung tissues, which subsequently allowed the transduction in the presence of pre‐existing anti‐AAV nAbs.

## 3 Discussion

AAV‐mediated gene therapies are a promising therapeutic modality with the potential to cure genetic diseases. However, due to the protein nature of their viral capsids and the intrinsic immune‐active property of their genomic nucleic acids, AAV‐based gene therapies usually induce the production of nAbs that not only reduce their transduction efficiency but disable their re‐dosing potential.^[^ ^1^, ^8^, ^18^, ^32^ ^]^ Although many efforts have been made to navigate the immunogenicity of AAVs, for example, capsid engineering,^[^ ^18^, ^20^, ^33^, ^34^ ^]^ genome engineering^[^ ^35^ ^]^ and co‐delivery with immunosuppressive agents,^[^ ^12^, ^36^ ^]^ overcoming the pre‐existing anti‐AAV immunity and evading neutralization by nAbs consistently remain a significant challenge. In this work, we presented a new approach, based on physical anchoring of AAVs to the surface of RBCs, which we refer to as Hitchhiking‐Enabled AAV Re‐dosing and Targeting (HEART), to overcome neutralization by pre‐existing anti‐AAV nAbs and, in turn, to enable repeated administration as well as targeted transduction in the lungs by AAV‐mediated gene therapies.

HEART is built upon the RBC hitchhiking technology that involves the immobilization of AAVs to the surface of RBCs by a polyphenol‐mediated method. The unique design of the HEART system allows for two functionalities based on physical mechanism: i) targeted transduction in the lung tissue and ii) navigation of the viral neutralization by nAbs to enable AAV re‐dosing in the presence of an anti‐AAV immunity. Fundamentally, the targeted transduction in the lung tissue is derived from the biophysics of the carrier RBCs in HEART. More specifically, in agreement with our previous findings,^[^ ^27^, ^28^, ^30^ ^]^ this work proved that nano‐sized particles, such as the AAV particles, are sheared‐off and deposited in the lungs as RBCs squeeze through the narrow lung capillaries. Impressively, the physical anchoring of AAVs to RBCs in HEART also reduces the neutralization of AAVs by nAbs that subsequently leads to the gene expression in the lung tissue. As demonstrated in our mechanistic studies, HEART did not affect the induction of the humoral and cellular immune responses as compared to the administration of free AAVs. Instead, HEART followed a novel diffusion‐based mechanism to limit interaction with nAbs. Specifically, the physical immobilization of AAV particles to a large area of surface (RBC membrane) reduces the probability of interaction between AAVs and nAbs, and thus sufficiently delays the neutralization process that in turn creates a time window for the deposition of AAVs to the lungs.

Of note, HEART is mechanistically distinct from the existing technologies for overcoming anti‐AAV immune responses and may represent a new strategy to cope with existing nAbs. A majority of the existing technologies to address AAV immunogenicity, such as engineering low‐immunogenic AAV capsids, are focused on inhibiting/reducing the elicitation of the anti‐AAV immune response.^[^ ^12^, ^18^, ^20^, ^33^, ^34^, ^36^ ^]^ These technologies are especially applicable to patients who do not have pre‐existing AAV nAbs that enables repetitive administrations to ensure long‐term gene modification activities. However, the presence of pre‐existing anti‐AAV nAbs is very prevalent, even in patients who have not been treated with AAV gene therapies.^[^ ^36^, ^37^ ^]^ Particularly, it has been reported that up to 50% of patients who need AAV gene therapies have pre‐existing antibodies.^[^ ^38^, ^39^, ^40^ ^]^ The presence of pre‐existing nAbs can render the patient ineligible for the AAV treatment. HEART offers a potential strategy to solve this challenge. Owing to its capability of reducing nAb neutralization, HEART may not only enable repetitive dosing of AAV gene therapies in patients without pre‐existing nAbs at the time of treatment, but it broadens the eligibility of patients to those with pre‐existing nAbs.

Previous studies have demonstrated that, by changing the injection sites, RBC hitchhiking can target nanoparticles to tissues immediately downstream of the injection vessels including brain and kidneys.^[^ ^26^ ^]^ Thus, while the studies reported here focus on lung targeting, HEART can potentially offer a generalized targeting strategy to enable AAV transduction in tissues apart from the lung by changing the injection location. The potential of HEART for clinical translation requires further investigation. Notably, transfusion of RBCs has been widely adopted in the clinic, and various AAV serotypes, AAV9, AAV8, and AAV2 in particular, have advanced to late‐stage clinical studies. The outstanding safety profiles of RBCs and AAVs render the HEART technology more clinically translatable. Our preliminary studies have demonstrated the biocompatibility of HEART. However, the impact of different AAV serotypes and their loading capacity on the delivery outcomes and biocompatibility of HEART will be further investigated. In this current work, we have used AAV9 as a model to evaluate HEART's capability for targeted transduction and enabling AAV re‐dosing. The applicability of HEART to other AAV serotypes needs to be investigated further in future studies. In addition, this present work is a proof‐of‐concept study to introduce HEART as a novel delivery technology of AAV using two model gene constructs that encode eGFP and fLuc. HEART's efficacy in the functional gene transfer and therapeutic outcomes in a disease model must also be investigated in future studies to demonstrate its clinical utility. Moreover, the impact of HEART on the long‐term expression of target genes following repeated administrations will also be investigated in future studies. Taken together, this RBC‐mediated approach (HEART) can be a new, promising strategy for repetitive, targeted AAV‐mediated gene therapy.

## 4 Experimental Section

### Materials

AAV9 with CMV promoter‐driven expression of eGFP (AAV‐eGFP) and with CMV‐IVS promoter‐driven expression of fLuc (AAV‐fLuc) were purchased from Vector Biolabs (PA, USA) and used as received. Tannic acid (TA), iron(III) chloride hexahydrate (FeCl_3_∙6H_2_O), and 96% ethanol laboratory reagent were purchased from Millipore Sigma (MO, USA). Antibodies for immune cell profiling assay were purchased from BioLegend (CA, USA) unless otherwise noted. ACK lysis buffer, phosphate‐buffered saline (PBS), Power SYBR Green PCR master mix, and GeneJet Genomic DNA and Whole Blood Genomic DNA Purification kits were purchased from Thermo Fisher Scientific (MA, USA). Primers were custom‐ordered through IDT (IA, USA). 200 nm carboxylated polystyrene beads were purchased from Polysciences (PA, USA). Xenolight‐D‐luciferin potassium salt was obtained from Perkin Elmer (MA, USA). Lithium heparin coated Microtainer tubes were obtained from BD Medical Technology (NJ, USA). 0.9% saline solution was obtained from Teknova (CA, USA).

### Animals

Female BALB/c mice (aged 7–8 weeks) and C57BL/6 mice (aged 7–8 weeks) were purchased from Charles River Laboratories (Wilmington, MA). All experiments were performed according to the approved protocols by the Institutional Animal Care and Use Committee (IACUC) of the Faculty of Arts and Sciences (FAS), Harvard University (protocol number 18‐02‐320‐1).

### Cell Culture

EA.hy926 human endothelial cells (ATCC, CRL‐2922) were cultured in a humidified incubator maintained at 37 °C and 5% CO_2_. They were cultured in DMEM media supplemented with 10% FBS and 1% Pen‐Strep.

### Blood Collection

Whole blood was collected from mice via cardiac puncture using heparin pre‐coated syringe and Microtainer tubes. Blood was centrifuged at 1000 *g* for 10 min at 4 °C to isolate pelleted RBCs. RBCs were washed three times by resuspending with cold PBS and centrifuging at 650 *g* for 15 min at 4 °C, and were finally resuspended at 10% hematocrit in PBS (RBC stock solution) until use. RBCs were freshly prepared for every experiment.

### Preparation and Characterization of RBC‐Anchored AAV

11.5–30 µL of AAV solution (2.5–4.4 × 10^13^ vg/mL) and 10 µL of tannic acid solution in water (10 mg mL^−1^) were added to 400 µL PBS, vortexed, and incubated for 60 s to allow interaction between AAV and polyphenols. Tannic acid was used as the polyphenol of choice based on previously described protocol as well as its compatibility with RBC.^[^ ^29^, ^41^ ^]^ 100 µL of RBC stock solution, 3 µL FeCl_3_ solution (10 mg mL^−1^ in water), and 500 µL of PBS were added to the mixture sequentially with pipette mixing to achieve the adsorption of AAV‐polyphenol nanocomplexes on RBCs. The AAV to RBC incubation ratio was determined to be 2300:1 and this ratio was chosen based on the AAV dose required for in vivo administrations. The product was washed three times by resuspending with cold PBS and centrifuging at 100 g for 5 min at 4 °C to remove any excess unbound materials. Approximately 10 µL (10% hematocrit of 100 µL RBC stock solution initially added) of AAV‐hitchhiked RBCs (RBC‐AAV) was resuspended in 90 µL PBS to achieve the 100‐µL injection‐ready solution.

Scanning electron microscopy (SEM) images were obtained on a ZEISS FESEM Ultra‐55 field‐emission scanning electron microscope (Carl Zeiss, Germany), operating at an accelerating voltage of ≈5–10 kV. RBC‐AAV samples were fixed using 2.5% glutaraldehyde solution, washed with an increasing ethanol gradient, chemically dried using hexamethyldisilazane and sputter‐coated (EMT 150T ES metal sputter coater, PA, USA) prior to imaging. TEM were performed on a JEOL JEM‐1400 TEM instrument operating at a voltage of 100 kV (JEOL US, Inc.) by air‐drying 2 µL of RBC‐AAV samples on Formvar carbon‐coated gold grids. Binding efficiency of AAV on RBCs was determined using RT‐qPCR. Total viral genome from injection‐ready RBC‐AAV solution was extracted using GeneJet Whole Blood Genomic DNA purification kit per manufacturer's instruction, which was quantified via RT‐qPCR using the standard curve generated with free AAV. Primers specific to the universal inverted terminal repeat (ITR) sequence of AAVs (Table S1, Supporting Information) were used, with PCR cycles as follows: 98 °C 3 min/98 °C 15 s/58 °C 30 s/repeat 39 times. Hemolysis of RBCs from the hitchhiking process was determined by measuring the absorbance of hemoglobin (405 nm) released from lysed RBCs via a plate reader (BioTek Synergy Neo2).

### In Vitro AAV‐eGFP Transduction

EA.hy926 human endothelial cells were seeded in a 6‐well plate at 200 000 cells/well, and incubated overnight prior to treatment. Cells were treated for 14 days with an equivalent dose of either free AAV‐eGFP or AAV‐eGFP recovered from RBC‐AAV‐eGFP by lysing RBCs at different AAV to endothelial ratios. eGFP expression in transduced cells was both visualized with fluorescence microscopy and quantified with flow cytometer (BD LSR II Analyser).

### Biodistribution in Mice without Neutralizing Antibodies (nAbs)

Female 7–8 weeks old C57BL/6 mice (*n* = 3 per group) were intravenously administered with the equivalent dose (3.8 × 10^10^ vg/mouse) of either free AAV‐eGFP or RBC‐AAV‐eGFP. Mice were sacrificed by CO_2_ overdose at 1 and 24 h after injection and major organs, including heart, lung, liver, spleen, kidneys and brain, were harvested and weighed. Organs were homogenized in water using a high shear homogenizer (IKA T 10 Basic ULTRA‐TURRAX, NC, USA) and viral genome copies in each organ were extracted from the same mass of organ homogenates (10 mg for liver, 5 mg for others) using a GeneJet Genomic DNA Purification kit per manufacturer's instruction. The amount of viral genome copies was quantified via RT‐qPCR using ITR‐specific primers and the standard curve generated with blank organ homogenates spiked in with free AAV. Percentage of injected AAVs accumulated in an organ was calculated based on the number of viral copies detected in an organ from qPCR following viral genome extraction and the total viral copies injected as determined from the binding efficiency.

### Biodistribution in Mice with AAV‐Specific nAbs

Ten female 7–8 weeks old C57BL/6 mice were intravenously administered with free AAV‐fLuc at 1 × 10^11^ vg/mouse. Three weeks after the initial AAV exposure, the same mice were intravenously injected with the equivalent dose (3.8 × 10^10^ vg/mouse) of either free AAV‐eGFP or RBC‐AAV‐eGFP. Mice were sacrificed by CO_2_ overdose at 2.5 min after injection and major organs were harvested. The same viral genome extraction and RT‐qPCR procedures were followed to determine the biodistribution, with the exception of eGFP‐specific primers in place of ITR‐specific primers (Table S1, Supporting Information).

### Single AAV Administration in Mice without nAbs

Female 7–8 weeks old BALB/c mice (*n* = 4 per group) were intravenously administered with the equivalent dose (2.6 × 10^10^ vg/mouse) of either free AAV‐fLuc or RBC‐AAV‐fLuc.

### Single AAV Administration in Mice with AAV‐Specific nAbs

Female 7–8 weeks old C57BL/6 mice were intravenously administered with the equivalent dose (3.8 × 10^10^ vg/mouse) of either free AAV‐eGFP or RBC‐AAV‐eGFP. Four weeks after the initial AAV exposure, the same mice were intravenously injected with the equivalent dose (3.8 × 10^10^ vg/mouse) of either free AAV‐fLuc or RBC‐AAV‐fLuc.

### Repeated AAV Administration in Mice

Female 7–8 weeks old C57BL/6 mice were intravenously administered with the equivalent dose (3.8 × 10^10^ vg/mouse) of either free AAV‐fLuc or RBC‐AAV‐fLuc. Four weeks after the initial AAV exposure, the same mice were intravenously injected with the equivalent dose (3.8 × 10^10^ vg/mouse) of either free AAV‐fLuc or RBC‐AAV‐fLuc.

### In Vivo Transduction Efficacy

Following the administration of either free AAV‐fLuc or RBC‐AAV‐fLuc in multiple studies, luciferase expression in live animals was monitored every 7 days following the injection by administering intraperitoneally with 150 µL of XenoLight‐D‐luciferin (30 mg mL^−1^ in saline) and imaging 15 min after using a Perkin Elmer IVIS small animal imaging system. The average radiance (bioluminescence intensity) was evaluated using the software Living system. On the day of the specified end‐point for each experiment, mice were injected intraperitoneally with D‐luciferin and euthanized 15 min after to extract and image organs ex vivo using IVIS.

### In Vivo Toxicity

Female 7–8 weeks old C57BL/6 mice were intravenously administered with the equivalent dose (3.8 × 10^10^ vg/mouse) of either free AAV‐fLuc or RBC‐AAV‐fLuc. Mice were euthanized at 1 and 7 days post‐injection, and blood and major organs were harvested. Organs were fixed in paraformaldehyde, sectioned for H&E staining, and interpreted by a professional histopathologist at the Rodent Histopathology Core in Harvard Medical School. Whole blood and serum were analyzed for comprehensive complete blood count and blood chemistry (IDEXX BioAnalytics, North Grafton, MA).

### Immune Cell Profiling

Different panels of antibody cocktails were generated from anti‐CD45 (BioLegend, 103116, 30‐F11), anti‐CD3 (BioLegend, 100218, 17A2), anti‐CD4 (BioLegend, 100421, GK1x.5), anti‐CD8a (BioLegend, 100711, 53‐6.7), anti‐NKp46 (BioLegend, 137606, 29A1.4), anti‐CD11c (BioLegend, 117307, N418), anti‐CD25 (Biolegend, 101908, 3C7), anti‐FOXP3 (Biolegend, 126404, MF‐14), anti‐granzyme B (BioLegend, 372208, QA16A02), anti‐IFN*γ* (BioLegend, 505849, XMG1.2), anti‐IFN*γ* (BioLegend, 505806, XMG1.2, anti‐CD62L (BioLegend, 104432, MEL‐14) and anti‐CD44 (BD Biosciences, 560568, IM7) antibodies, and the AmCyan Live/Dead Cell Staining Kit (Thermo Fisher Scientific). All antibodies were diluted at optimized dilutions before being used.

Female 7–8 weeks old C57BL/6 mice (*n* = 4–5 per group) were intravenously administered with the equivalent dose (3.8 × 10^10^ vg/mouse) of either free AAV‐fLuc or RBC‐AAV‐fLuc. Mice were then euthanized on day 31, and spleen and lungs were collected. Organ dissociation kits (Miltenyi Biotec) were used per manufacturer's instruction to generate a single‐cell suspension from excised organs, and cells were stained with the antibodies listed above and analyzed using flow cytometry (BD LSR II Analyser). Flow cytometry data analyses were performed using FlowJo 10 software.

### nAb Titration from AAV Administration

Female 7–8 weeks old C57BL/6 mice (*n* = 4 per group) were intravenously administered with the equivalent dose (3.8 × 10^10^ vg/mouse) of either free AAV‐eGFP or RBC‐AAV‐eGFP. Four weeks after the initial AAV exposure, the same mice were intravenously injected with the equivalent dose (3.8 × 10^10^ vg/mouse) of either free AAV‐fLuc or RBC‐AAV‐fLuc. Blood was collected on days 0, 27, 42, and 56 post the first injection to quantify the amount of nAbs. nAb titer was measured by ELISA. In brief, 96‐well plate was coated with AAV‐eGFP (2.5 × 10^9^ vg/well in 100 µL PBS) overnight at 4 °C. Plates were then washed 6 times with washing buffer (PBS + 0.01% Tween‐20) and blocked using 300 µL of Pierce Protein‐Free (PBS) Blocking Buffer (Thermo Scientific) for 2 h. After washing the plates 6 times, 100 µL of serum at a serial dilution was added to each well and incubated for 2 h at room temperature. The plates were then washed again and goat anti‐mouse IgG HRP in 100 µL of PBS (1:10 000 dilution) was added to each well for incubation for 1 h. After washing the plates, 100 µL TMB substrate was added to each well; 10 min later, the reaction was stopped by adding 100 µL stopping buffer. Absorbance at 450 nm was measured using a plate reader. The antibody titer (EC_50_) was defined as the dilution factor at which the absorbance value dropped to half of the maximum.

### Modeling of Interaction between nAb and RBC‐AAV

Brownian diffusion modeling is used to provide a quantitative estimate of the reduction in interaction probabilities between nAb and RBC‐immobilized AAV. Assuming AAVs and nAbs as spheres of radius *R*_AAV_ and *R*_nAb_, respectively, the time evolution of the probability of spheres to interact with each other starting from a separation of *l* can be written as:

(1) $$p\left(t\right)=\xi \frac{R_{\mathrm{AAV}}+R_{\mathrm{nAb}}}{l}\frac{\Delta l}{\sqrt{4\pi D}t^{3/2}}e^{\frac{\Delta l^{2}}{4Dt}}$$

where *p* is the probability of interaction, Δl = l − (*R*_AAV_ + *R*_nAb_) is the total distance the species have to diffuse before interacting with each other, *D* is the total diffusivity of the system, and *t* is time. *ξ* is a site accessibility factor that physically captures the surface fraction of the AAV that is available for binding with nAbs, and is equal to 1 for free AAV's and is equal to 0.5 for immobilized AAV's. MATLAB was used to generate the plot of analytical solutions to the obtained governing equation. To show the effect of the initial separation we varied the initial separation by 50% from a baseline case of *l*_aav;im_ = 1000 (*R*_aav_ + *R*_nab_). For the case where the AAVs are free, the statistically most likely separation *l*_aav;m_ is equal to 0.75*l*_aav;im_.

### In Vitro Binding Study

Monoclonal anti‐AAV9 antibody (Millipore Sigma, MO, USA) was labeled with Alexa Fluor 647 (Thermo Fisher, Waltham, MA, USA), and the resulting protein concentration was measured by NanoDrop One. Either free AAV‐fLuc or RBC‐AAV‐fLuc at equivalent AAV concentration in 1× PBS (3.8 × 10^10^ vg) was incubated with anti‐AAV9 antibodies (20 ng; approximately equimolar to AAV) for 15 min. Free AAV sample was filtered through a 300 kDa MWCO Nanosep filter (Pall, NY, USA), and fluorescence of the filtrate was measured by plate reader. RBC‐AAV sample was centrifuged at 100 g for 5 min, and fluorescence of the supernatant was measured by plate reader. The fluorescence intensity values were normalized to that of free antibodies of the same initial concentration to determine the percentage of unbound antibodies in each sample.

### Statistics

All data are presented as mean ± sem. Statistical analyses were performed using GraphPad Prism 8 software. Comparison between two groups was conducted using unpaired two‐tailed Student's *t* test, and comparisons among multiple groups were conducted using one‐way analysis of variance (ANOVA) followed by HST test. *p* values represent levels of significance: *p* < 0.001 ***, *p* < 0.01 **, and *p* < 0.05 *.

## Conflict of Interest

S.M. is a shareholder and board member of Hitch Bio. Z.Z., J.G., N.K., Y.G., and S.M. are inventors on patent applications licensed to Hitch Bio.

## Supporting information

Supporting Information

## Data Availability

The data that support the findings of this study are available in the supplementary material of this article.
