# Supplementary material for: Red Blood Cell Anchoring Enables Targeted Transduction and Re‐Administration of AAV‐Mediated Gene Therapy
Source: Adv Sci (Weinh). 2022 Jul 3;9(24):2201293. doi: 10.1002/advs.202201293 (PMC9404386; doi:10.1002/advs.202201293)
Supplement: Supplementary file 1 — Supporting Information [file ADVS-9-2201293-s001.pdf]

ADVANCED SCIENCE
Open Access

Supporting Information

for *Adv. Sci.*, DOI 10.1002/advs.202201293

Red Blood Cell Anchoring Enables Targeted Transduction and Re-Administration of AAV-Mediated Gene Therapy

*Zongmin Zhao, Jayoung Kim, Vinny Chandran Suja, Neha Kapate, Yongsheng Gao, Junling Guo, Vladimir R. Muzykantov and Samir Mitragotri**

Supporting Information

Red Blood Cell Anchoring Enables Targeted Transduction and Re-Administration of AAV-Mediated Gene Therapy

*Zongmin Zhao, Jayoung Kim, Vinny Chandran Suja, Neha Kapate, Yongsheng Gao, Junling Guo, Vladimir R. Muzykantov, Samir Mitragotri**

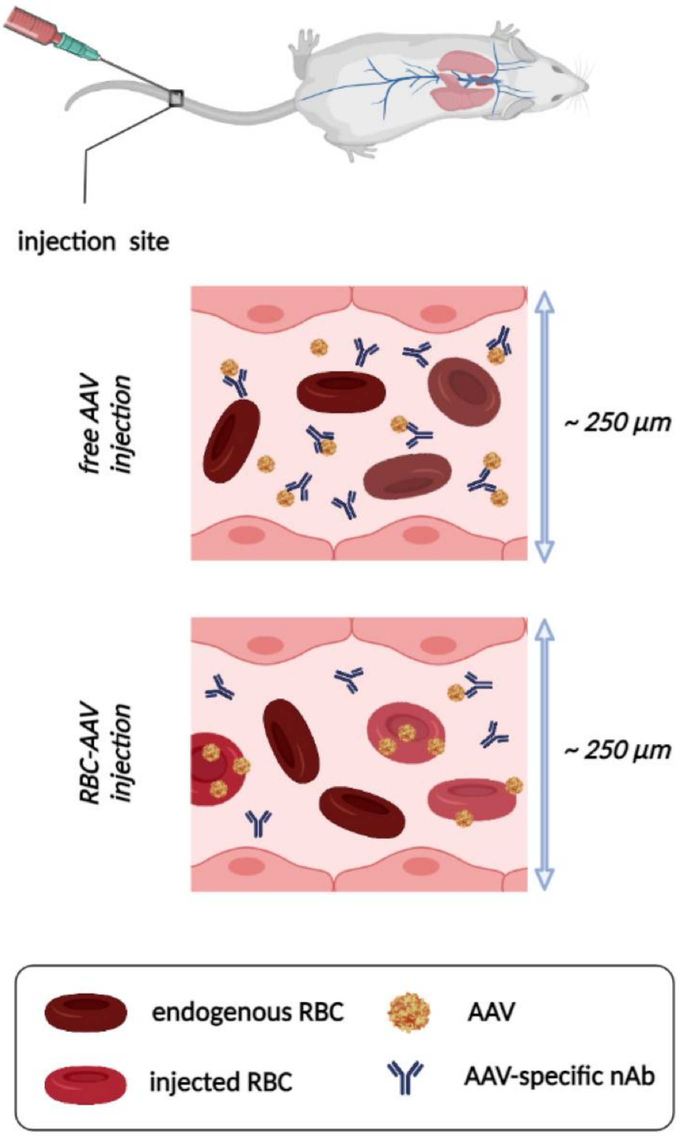

Figure S1. Schematic of free AAVs and RBC-AAVs in the tail-vein immediately after injection.

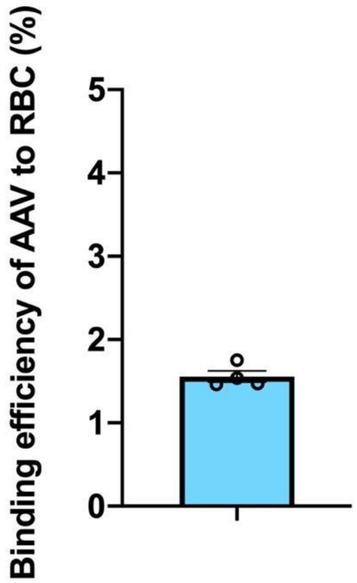

Figure S2. Natural binding of AAV onto RBCs. Binding efficiency of AAV onto RBCs. Data is presented as mean ± sem.

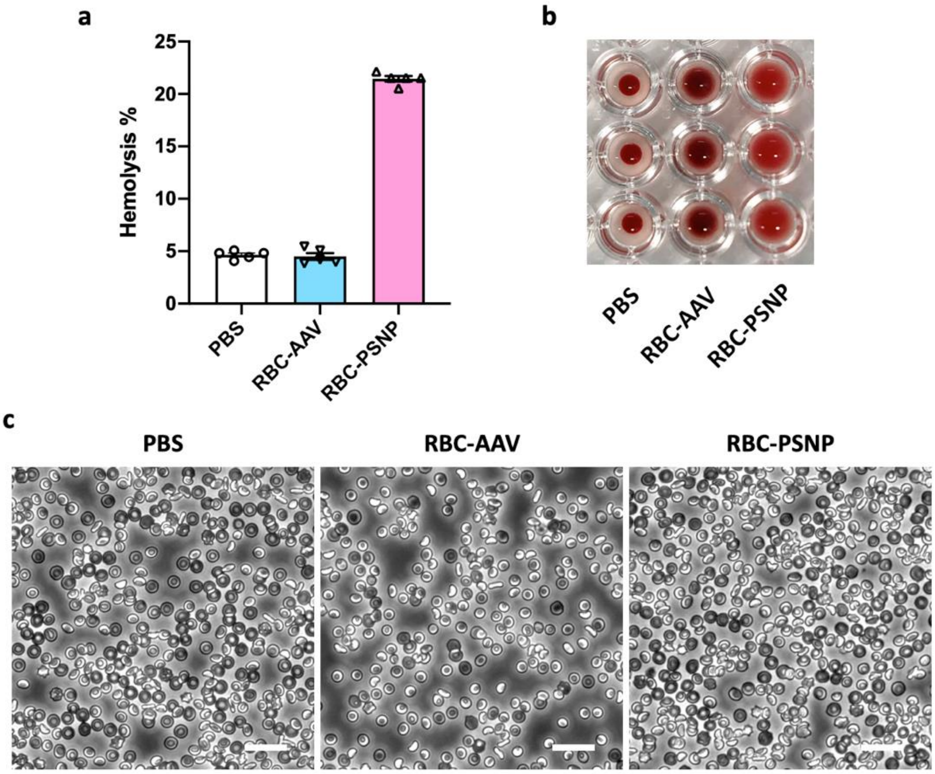

Figure S3. Impact of the anchoring of AAV onto RBCs by the polyphenol-mediated method on the carrier RBCs. (a) Percent hemolysis following the different processing of the RBCs. (b) Agglutination of RBCs after anchoring by AAVs. (c) Representative bright field images showing the morphology of RBCs after different processing. Scale bars represent 15 μm. In these studies, 200 nm polystyrene nanoparticles (RBC-PSNP) that can cause RBC damages were used a positive control.

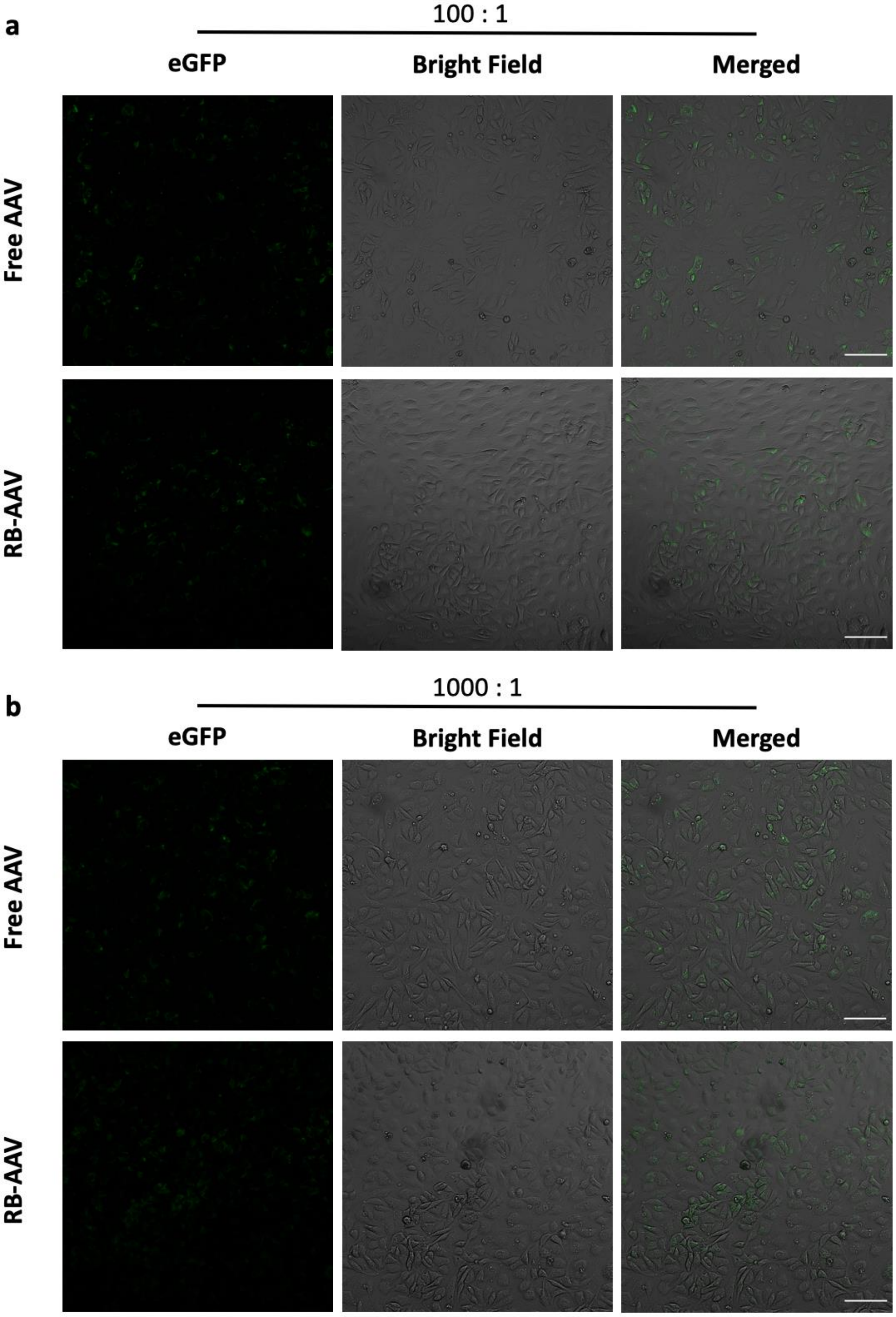

Figure S4. Transduction and eGFP expression in EA.hy926 endothelial cells by AAV-eGFP. Eqivalent dose of free AAV-eGFP (Free AAV) or AAV-eGFP detached from RBCs by lysing RBCs (RBC-AAV-eGFP) was incubated with EA.hy926 cells at a (**a**) 100:1 or (**b**) 1000:1 AAV to EA.hy926 cell ratio for 14 days. eGFP expression on day 14 was measured using confocal fluorescence microscopy. Scale bars represent 100 μm.

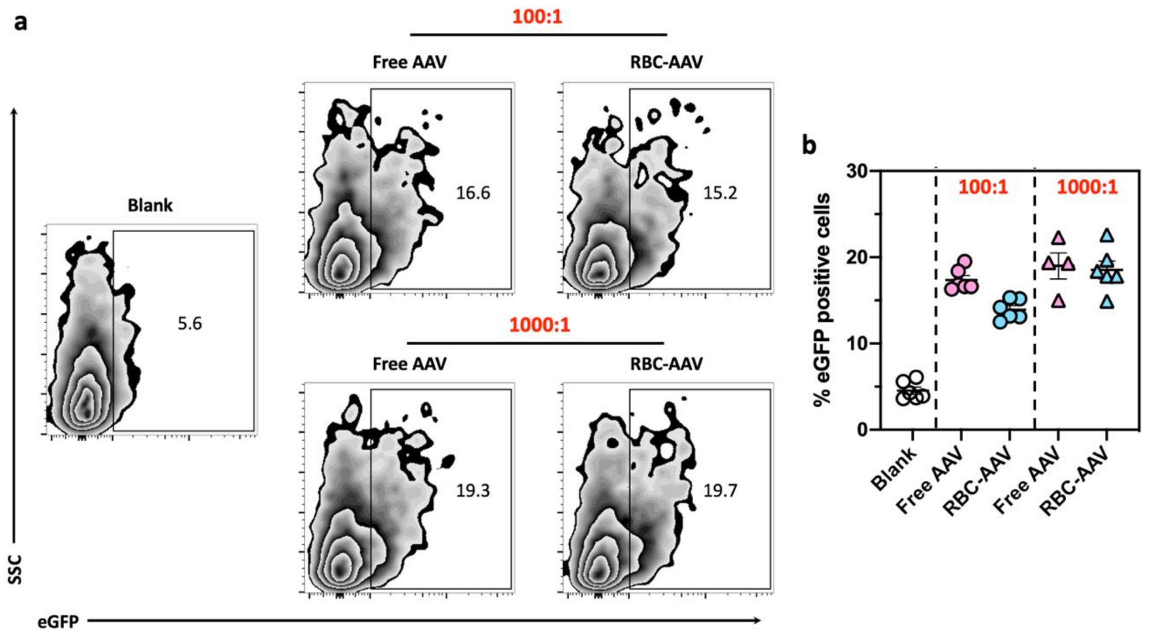

Figure S5. Expression of eGFP in EA.hy926 endothelial cells transfected by AAV-eGFP assayed by flow cytometry. (**a**) Representative flow plots showing the expression of eGFP in EA.hy926 cells. (**b**) Percent eGFP positive cells. In this study, equivalent dose of free AAV9-eGFP (Free AAV) or AAV-eGFP detached from RBCs by lysing RBCs (RBC-AAV-eGFP) was incubated with EA.hy926 cells at a 100:1 or 1000:1 AAV to EA.hy926 cell ratio for 14 days. eGFP expression on day 14 was measured using flow cytometry.

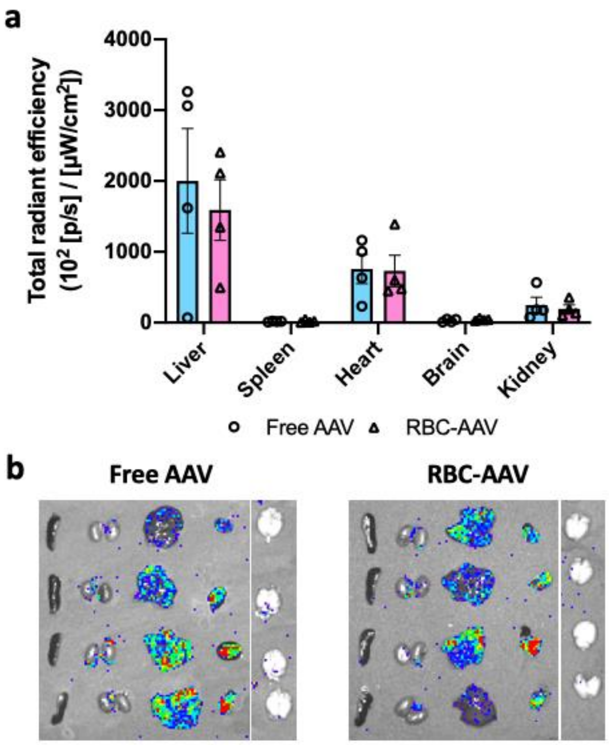

Figure S6. Luciferase expression in organs following a single-dose intravenous administration of AAV-fLuc formulations using the schedule shown in Figure 3b. (**a**) Quantification of the luciferase gene expression on day 40 as indicated by bioluminescence. (**b**) IVIS images of mouse organs on day 40 following the administration of AAV-fLuc formulations.

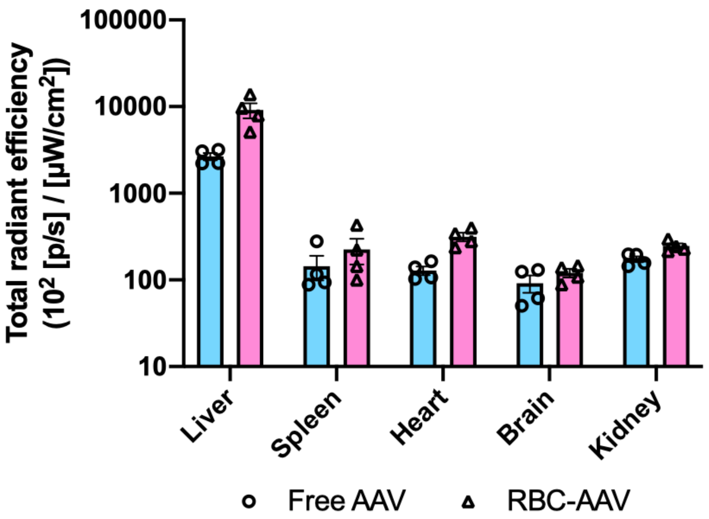

Figure S7. Luciferase expression in organs following a dual-dose administration of AAV-fLuc formulations using the schedule shown in Figure 3f. Luciferase gene expression in mouse organs on day 59 was quantified by bioluminescence using IVIS.

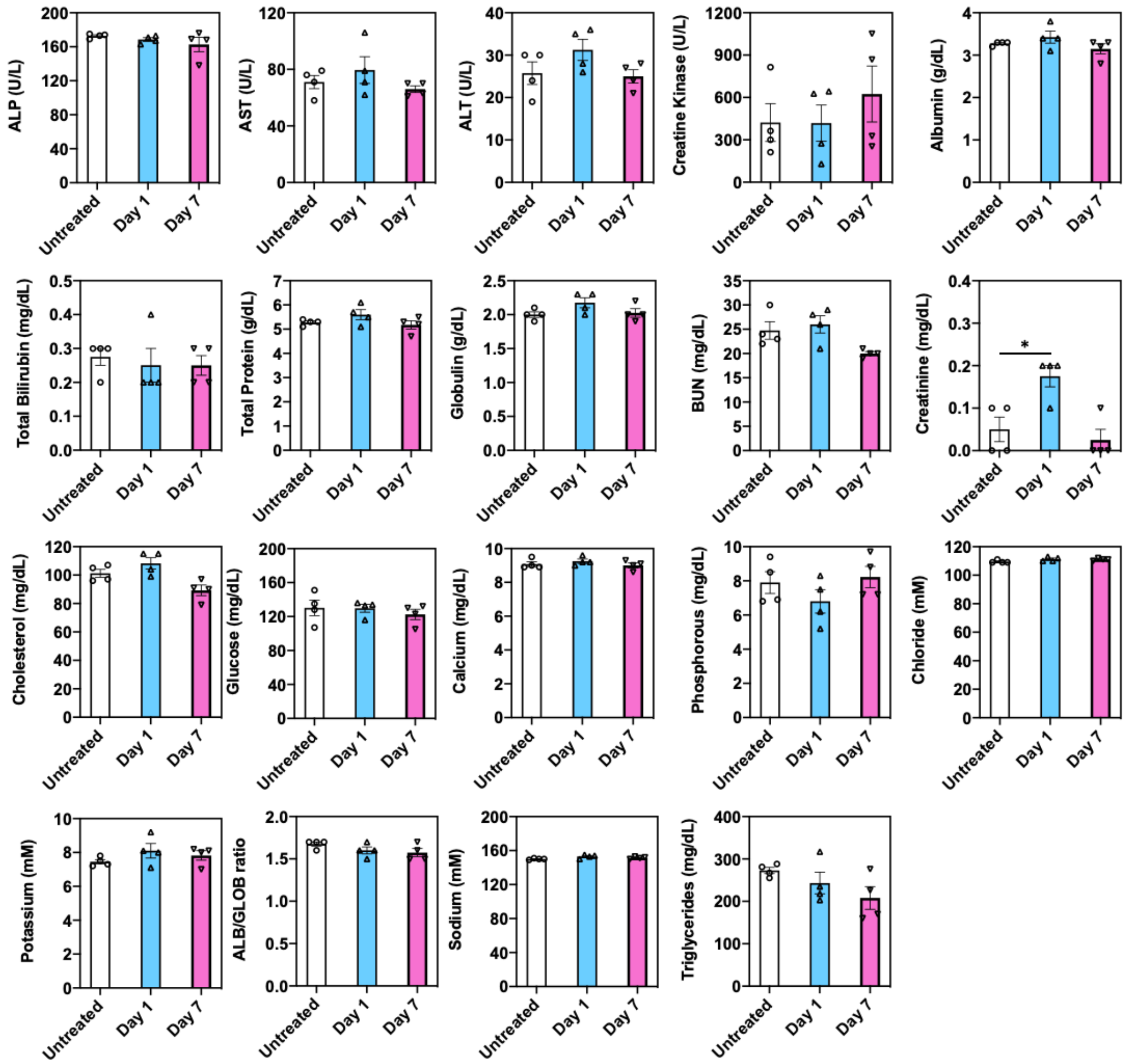

Figure S8. Serum chemistry of mice after intravenous administration of HEART. The HEART formulation was injected intravenously to mice; different serum chemistry parameters were measured on Day 1 and 7. No significant differences between the Untreated group (healthy mice without formulation administration) and the HEART group were detected for most of the tested serum chemistry parameters (One-way ANOVA followed by Tukey's HSD test) except for the creatinine level. While the creatinine level was significantly higher on Day 1 as compared to the Untreated group, it returned to normal on Day 7. Significantly different (One-way ANOVA followed by Tukey's HSD test): * $p < 0.05$.

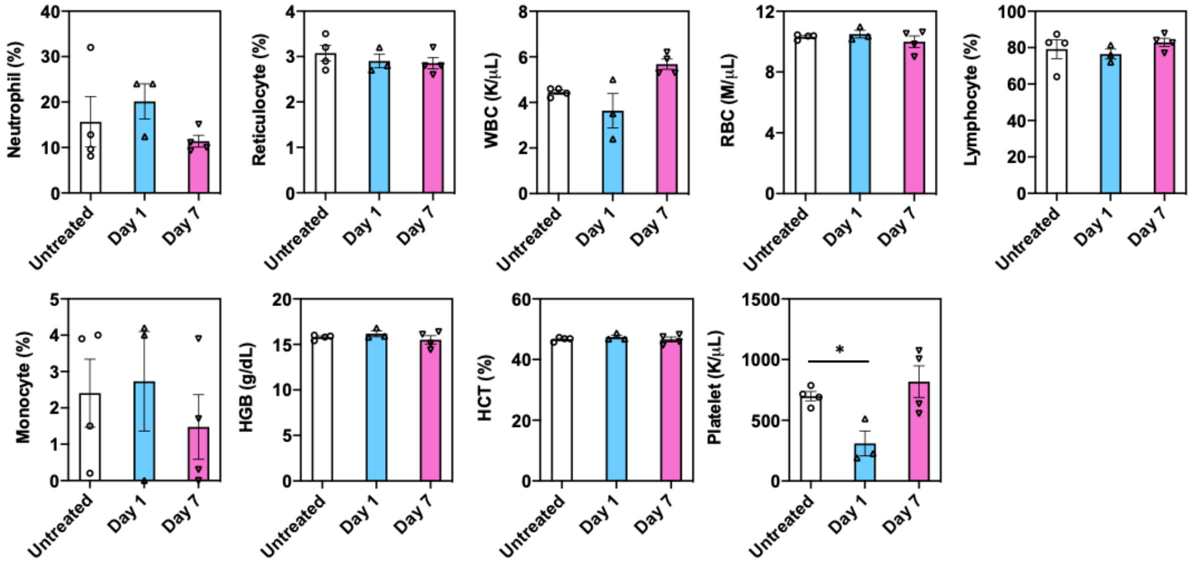

Figure S9. Hematological analysis of mice after intravenous administration of HEART. HEART formulation was injected intravenously to mice; different hematological parameters were measured on Day 1 and 7. No significant differences (One-way ANOVA followed by Tukey's HSD test) between the Untreated group and the HEART group were detected for most of the hematological parameters except for the platelet number. While the platelet number was significantly higher on Day 1 in the HEART group as compared to the Untreated group, it returned to normal on Day 7. Significantly different (One-way ANOVA followed by Tukey's HSD test): * $p < 0.05$.

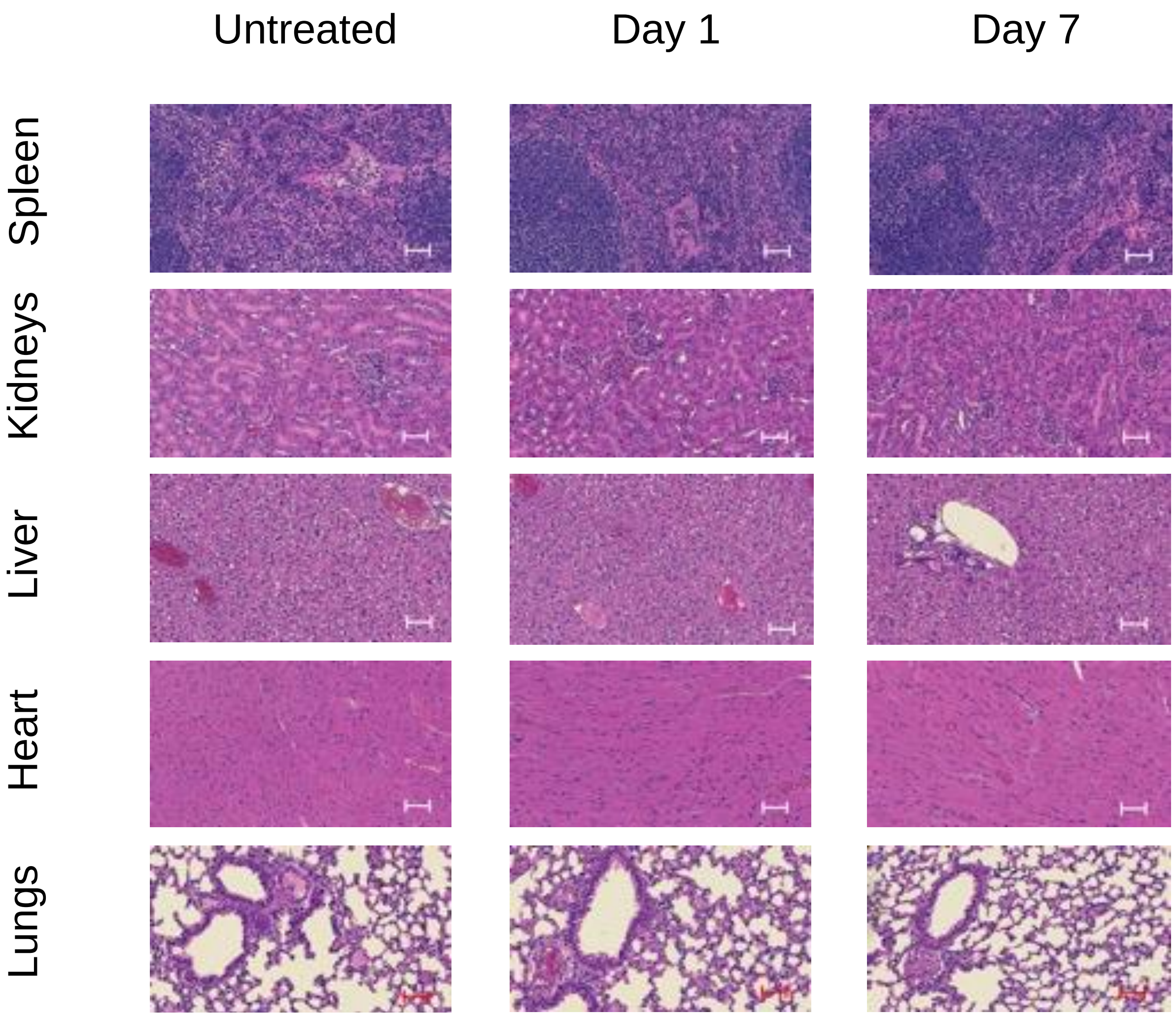

Figure S10. Representative H&E staining images of major mouse organs 1 and 7 days after intravenous administration of HEART (scale bar: 50 μm).

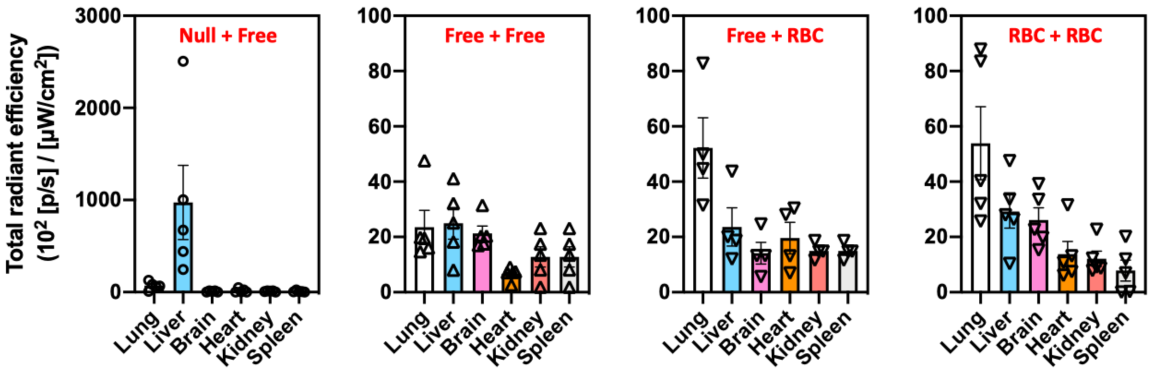

Figure S11. Luciferase expression in organs 37 days after the 2nd dose of AAV formulations using the schedule shown in Figure 3f. Luciferase gene expression in mouse organs on day 59 was quantified by bioluminescence using IVIS.

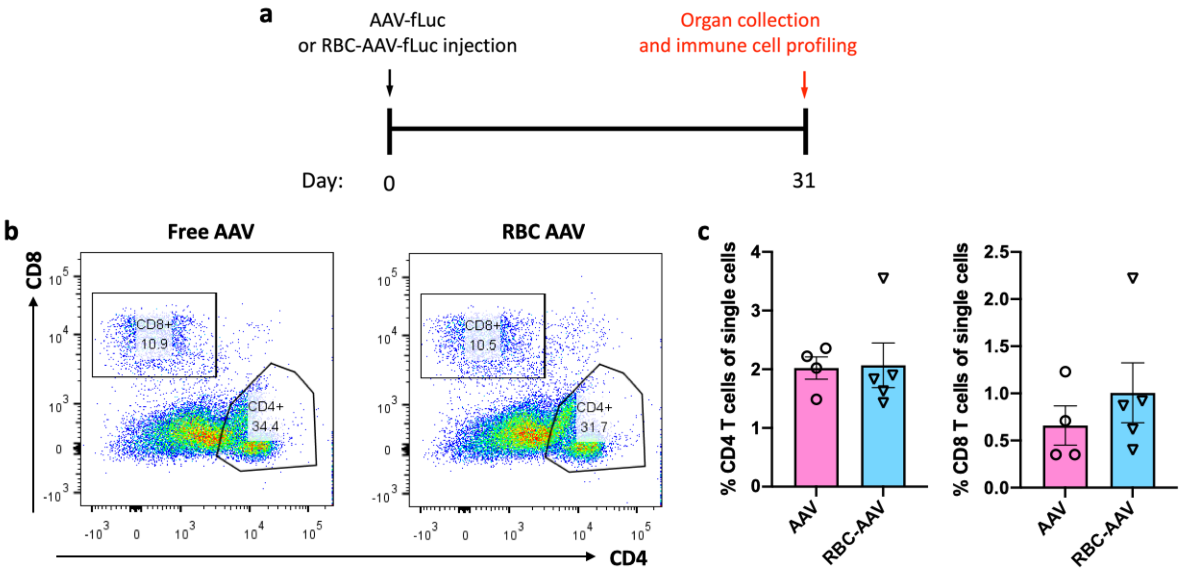

Figure S12. Immune cell profiles in the lungs following the administration of different AAV formulations. (**a**) Schematic showing the schedule of the immune cell profiling study. (**b**) Representative flow cytometry plots of CD4+ and CD8+ cells gated on CD45+CD3+ single cells. (**c**) The number of CD4 and CD8 T cells in the lung tissue. No significant differences were detected between the free AAV and RBC-AAV group in (**c**) using two-tailed student's t test.

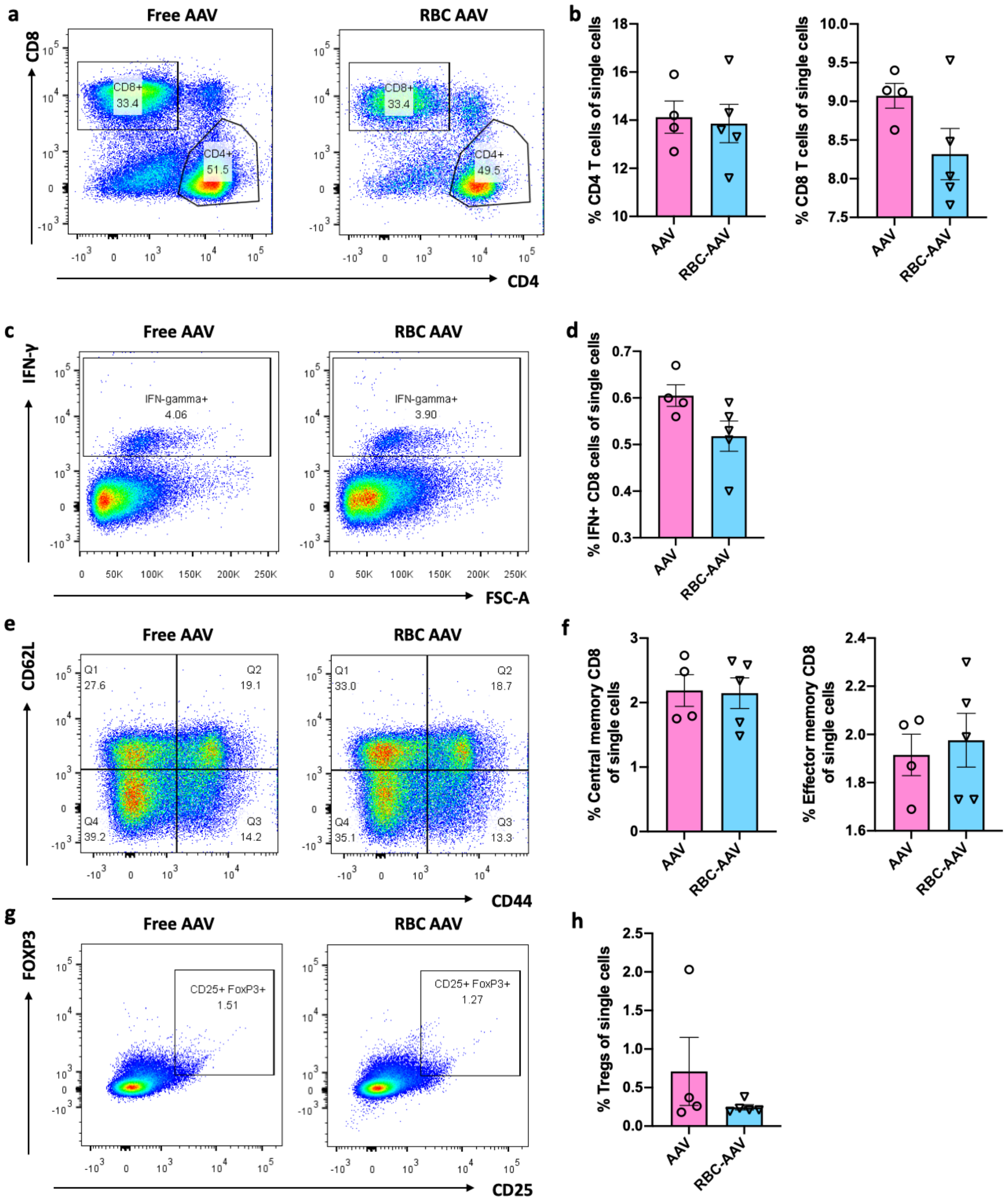

Figure S13. Immune cell profiles in the spleen following the administration of different AAV formulations. (**a**) Representative flow cytometry plots of CD4+ and CD8+ cells gated on CD45+CD3+ single cells. (**b**) The number of CD4 and CD8 T cells in the spleen. (**c**) Representative flow cytometry plots of IFN-γ+ cells gated on CD8+ single cells. (**d**) The number of IFN-γ+ CD8 T cells in the spleen. (**e**) Representative flow cytometry plots of central memory (CD44+CD62L+) and effector memory (CD44+CD62L-) T cells gated on CD8+ single cells. (**f**) The number of central memory (CD44+CD62L+) and effector memory (CD44+CD62L-) CD8 T cells in the spleen. (**g**) Representative flow cytometry plots of Treg (CD25+FOXP3+) cells gated on CD3+CD4+ single cells. (**h**) The number of Treg (CD25+FOXP3+) cells in the spleen. No significant differences were detected between the free AAV group and the RBC-AAV group in (**b, d, f, h**) using two-tailed student's t test.

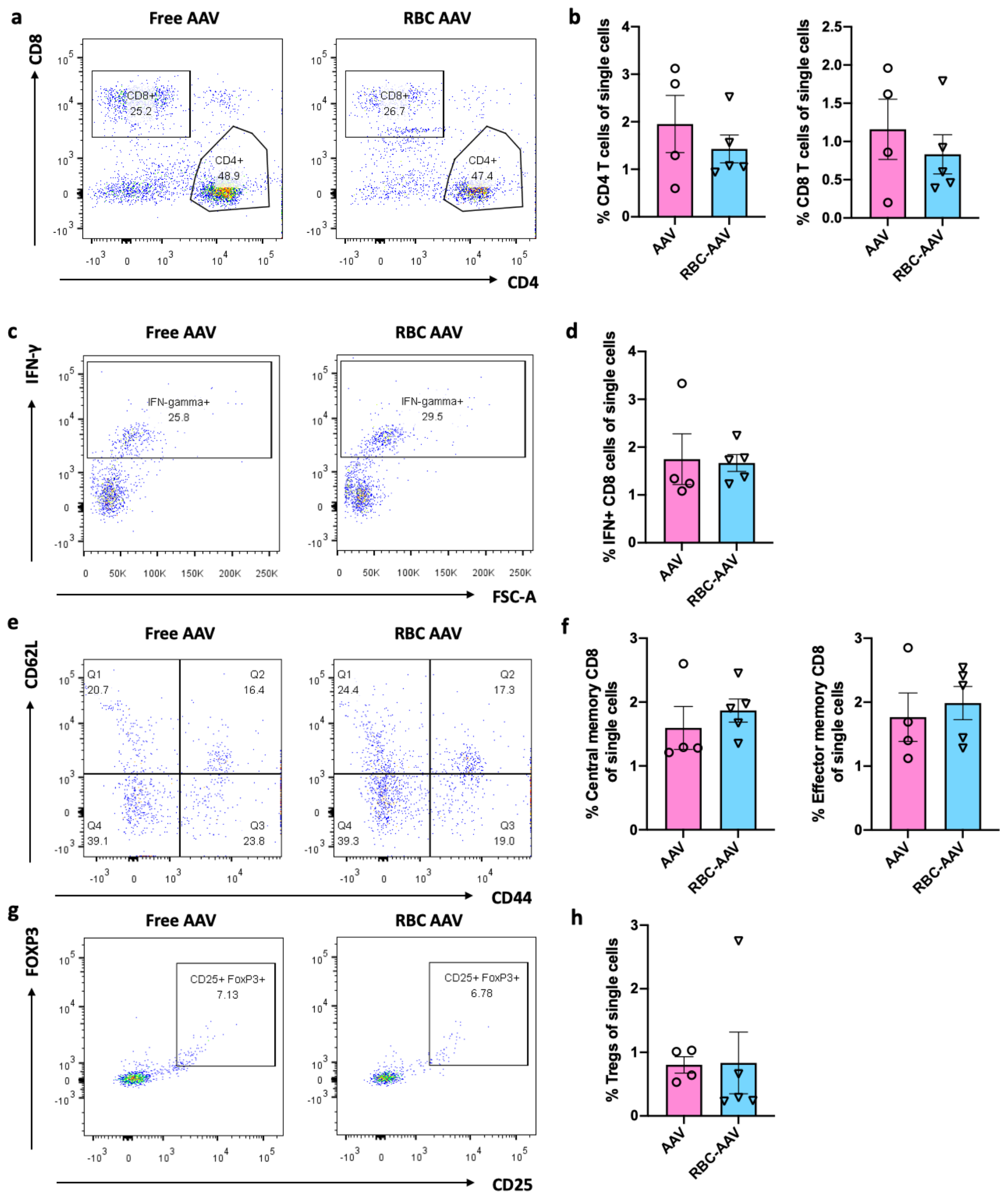

Figure S14. Immune cell profiles in the blood following the administration of different AAV formulations. (**a**) Representative flow cytometry plots of CD4+ and CD8+ cells gated on CD45+CD3+ single cells. (**b**) The number of CD4 and CD8 T cells in the blood. (**c**) Representative flow cytometry plots of IFN-γ+ cells gated on CD8+ single cells. (**d**) The number of IFN-γ+ CD8 T cells in the blood. (**e**) Representative flow cytometry plots of central memory (CD44+CD62L+) and effector memory (CD44+CD62L-) T cells gated on CD8+ single cells. (**f**) The number of central memory (CD44+CD62L+) and effector memory (CD44+CD62L-) CD8 T cells in the blood. (**g**) Representative flow cytometry plots of Treg (CD25+FOXP3+) cells gated on CD3+CD4+ single cells. (**h**) The number of Treg (CD25+FOXP3+) cells in the blood. No significant differences were detected between the free AAV group and the RBC-AAV group in (**b, d, f, h**) using two-tailed student's t test.

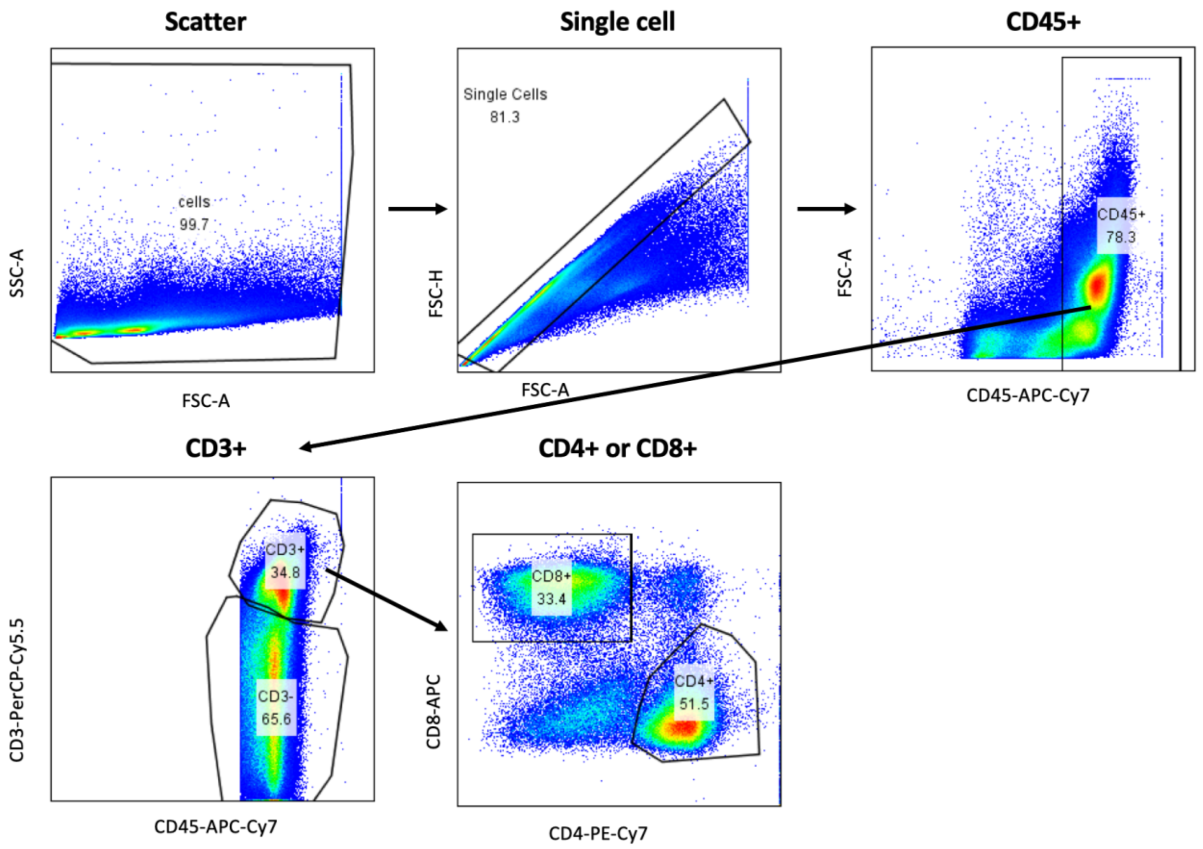

Figure S15. Representative flow cytometry gating strategies for CD4 and CD8 T cells shown in Fig. S12-14.

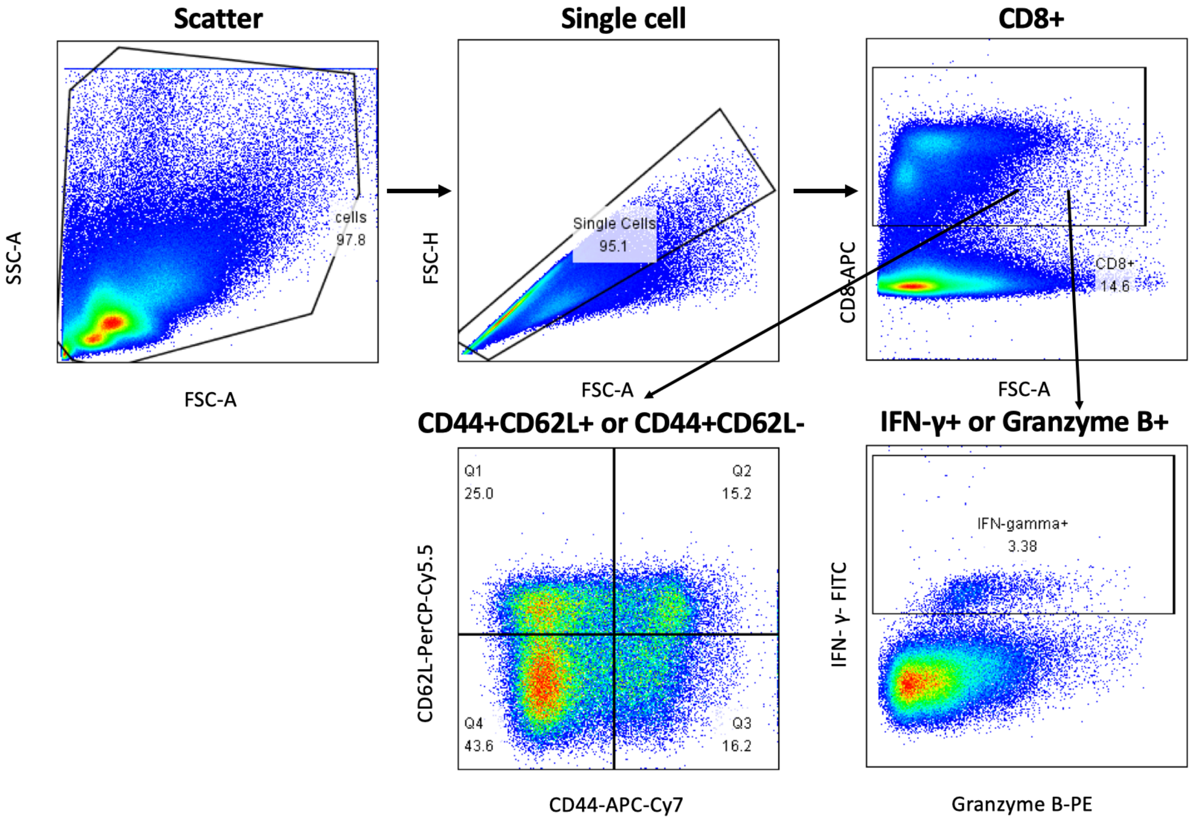

Figure S16. Representative flow cytometry gating strategies for IFN-γ+ effector CD8 T cells and memory T cells shown in Fig. S13-14.

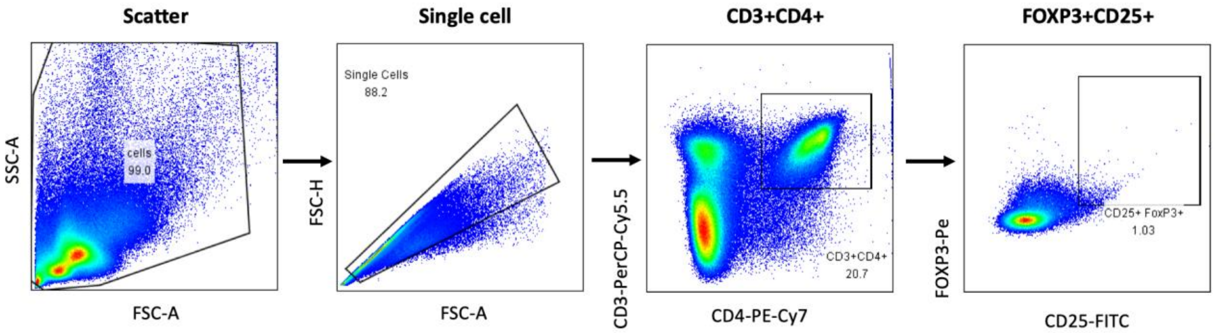

Figure S17. Representative flow cytometry gating strategies for FOXP3+CD25+ Treg cells shown in Fig. S13-14.

Table S1. Sequence for primers used in RT-qPCR.

**Primers**	**Forward**	**Reverse**
ITR	TCTAGTTGCCAGCCATCTGTTGT	TGGGAGTGGCACCTTCCA
eGFP	GACGACGGCAACTACAAGA	GATGCCCTTCAGCTCGAT
